# The Impact of Caring and Killing on Physiological and Psychometric Measures of Stress in Animal Shelter Employees: A Pilot Study

**DOI:** 10.3390/ijerph17249196

**Published:** 2020-12-09

**Authors:** Allison Andrukonis, Nathaniel J Hall, Alexandra Protopopova

**Affiliations:** 1 Department of Animal and Food Sciences, Texas Tech University, Lubbock, TX 79409, USA; Nathaniel.J.Hall@ttu.edu; 2 Department of Land and Food Systems, University of British Columbia, Vancouver, BC V6T 1Z4, Canada; a.protopopova@ubc.ca

**Keywords:** animal-care employees, animal shelter, compassion fatigue, occupational stress

## Abstract

Animal shelter employees are in a unique position where they care for, and later kill, the same animals. The aim of our exploratory study was to assess whether “caring” and/or “killing” evokes physiological and psychometric indicators of stress in employees. Experiment 1 compared three careers that kill regularly, but involve varying degrees of husbandry (*n* = 28). Blood pressure (BP), salivary cortisol, heart rate (HR), and heart rate variability (HRV) were collected; data showed higher HR and lower HRV during the process of killing. Psychometric scales showed that burnout and Impact Event Scale-Revised (IES-R) scores were higher in careers with higher contact with animals. Experiment 2 compared three careers that involve husbandry, but varying exposure to killing (*n* = 41). BP, cortisol awakening response, HR, and HRV were measured as well as Professional Quality of Life Scale, IES-R, and Moral Injury Event Scale were administered. There were no significant differences across careers in any measures. The data suggest that the process of killing may be physiologically stressful to the person, and higher levels of animal contact in a euthanasia context may be associated with burnout and traumatic stress, but that the act of euthanasia is not a unique predictor of overall occupational distress.

## 1. Introduction

Animal shelter employees euthanize approximately 1.5 million animals annually [1]. While some of the animals have significant health problems that may warrant a humane death, others are physically and behaviorally healthy. Animal shelter employees are in a unique position where they must provide husbandry for and subsequently euthanize the animals in their care. Arnold Arluke coined the phrase caring–killing paradox to highlight this specific challenge of caring for and later killing, in the animal shelter environment [2]. A recent study suggested that annual incidence of Post-Traumatic Stress Disorder (PTSD) in animal shelter employees is five times higher than the national average [3]. Along with PTSD, animal shelter employees often suffer from secondary traumatic stress (STS) [4]. STS is the reaction occurring in caregivers resulting from prolonged exposure to the stress associated with helping or wanting to help traumatized or suffering victims and differs from PTSD only in actual exposure to trauma [5,6].

Occupational stress is thought to be an important contributor to animal control officers, along with others in protective service occupations (e.g., police officers), having the highest workplace suicide rate, excluding military personnel, at 5.3 per 1 million people [7]. One hypothesized cause for such a high suicide rate in animal control workers is the caring–killing paradox [8]. Animal shelter employees primarily enter the job because of their love of animals [9]. However, the reality of their job is often more bleak than expected [10]. The contradiction between job expectations and the reality of having to kill can lead to moral stress [11,12]. Moral stress occurs when a person engages in, bears witness to, fails to prevent, or learns about acts that transgress that individual’s moral code [13]. Moral stress can lead to moral injury when that individual is unable to contextualize the experience and fit the actions into a pre-existing moral schema [13]. In the first empirical evaluation of moral injury in animal shelter employees, Andrukonis and Protopopova (2020) found that moral injury was likely present in animal shelter employees and was significantly higher in individuals who euthanize. Further, Reeve and colleagues (2005) found that animal shelter employees directly involved in euthanasia reported significantly higher levels of work stress, stress-related somatic complaints, and lower levels of work satisfaction compared to animal shelter employees not involved in euthanasia. This supports the argument that actual killing is an important precursor of occupational stress in animal-care employees.

Participation in euthanasia activities has also been associated with higher incidence of burnout [10,14,15,16]. In a 2015 survey of animal shelter managers, Anderson et al. found that nearly 75% of managers strongly believed that participating in euthanasia activities contributed to burnout. While common in animal shelter employees, burnout has been reported in a variety of animal-care careers [15,17,18,19,20]. Burnout, defined by three dimensions: exhaustion, cynicism, and inefficacy, is the result of chronic emotional and interpersonal stressors [21]. The reported high incidence of burnout in animal-care employees is not surprising given the emotional pull related to both caring for and killing animals [22,23,24].

Exposure to euthanasia has also been correlated with higher incidence of compassion fatigue [8,15,25]. Compassion fatigue was originally thought of as a unique form of burnout experienced exclusively in caring professions [26]. While compassion fatigue has been conceptualized in the human-care professions, animal-care professions have been left out [27]. For the purpose of this paper, we will refer to compassion fatigue as a state of emotional dysregulation, comprising secondary traumatic stress and burnout, that negatively influences individuals in caring professions [28,29]. Compassion satisfaction, or the gratification one gets from their work helping others, has been suggested to be a protective factor against developing compassion fatigue, burnout, and secondary traumatic stress [29,30,31]. Higher compassion satisfaction in animal-care workers has been correlated with increased human–animal interactions at work [17]. This suggests that providing care for animals may be a protective factor.

However, research in veterinarians has found that knowing the animal prior to euthanasia makes the process more stressful and that the degree of attachment to the animal may be an important antecedent of euthanasia-related stress [32]. This contradicts the protective nature of caring and suggests that animal shelter employees involved in animal care may experience higher levels of euthanasia-associated stress. There has been little research focused on whether providing care for an animal that later faces euthanasia increases the risk of negative occupational outcomes—perhaps because it is assumed that all animal-care work involves husbandry and, therefore, attachment to animals is inevitable [33]. In fact, Arluke found that animal shelter employees could not conceive the notion of not developing attachments to the animals in their care and that shelter employees found those attachments to be an important part of the job [33].

There are several physiological measures indicative of occupational stress, such as heart rate, heart rate variability, blood pressure, and cortisol [34,35,36]. During a stressful event, two systems are triggered: the autonomic nervous system and the hypothalamic-pituitary-adrenal (HPA) axis [37]. Heart rate variability (HRV) has been used as a measure of the autonomic nervous system during psychoemotional stress [38]. Defined as the beat-to-beat variation in heart rate, lower HRV has been associated with stress and PTSD [39,40]. The activation of the sympathetic nervous system leads to the release of epinephrine, which increases heart rate (HR) and blood pressure (BP). Following a stressor, the hypothalamus releases corticotropin-releasing hormone (CRH) [41]. CRH then stimulates the pituitary gland to release adrenocorticotropic hormone (ACTH), which then moves to adrenal glands, stimulating the release of cortisol. Increased cortisol levels have been associated with burnout [42]. Interestingly, lower cortisol levels at the time of trauma have been shown to predict the development of PTSD after 6 weeks and 6 months [43].

The aim of the current two-part exploratory study was to evaluate whether both caring for and killing animals are necessary for the expression of physiological and psychometric indicators of stress in animal-care employees. We expected both the husbandry (Experiment 1) and killing (Experiment 2) aspects of animal-care work to be significant in eliciting physiological indicators of stress and developing burnout, secondary traumatic stress, and moral injury. Using both a quasi-experimental group and within-subject design, physiological and psychometric data were collected on animal-care professions with (1) varying degrees of “caring” or husbandry duties: animal shelter employees, veterinarians, and university personnel who harvest livestock; and (2) exposure to killing: animal shelter employees who euthanize frequently, animal shelter employees who do not euthanize, and pet hotel employees. We hypothesized that PTSD indicators, burnout, secondary traumatic stress, and moral injury would be highest in animal shelter employees who euthanize regularly. We also hypothesized that compassion satisfaction would be lowest in animal shelter employees who euthanize regularly. We expected to see a decrease in HRV during killing and an increase in BP following killing. We also expected to see the greatest decrease in salivary cortisol in university personnel who harvest livestock.

## 2. Materials and Methods

The purposes of Experiment 1 and Experiment 2 were to evaluate “caring” (i.e., husbandry) and “killing” as predictors of physiological and psychological stress. Institutional Review Board approval was obtained from Texas Tech University (IRB2016-1014) for both experiments.

### 2.1. Experiment 1

#### 2.1.1. Participants

A convenience sample of sixteen animal shelter employees who currently euthanize, four veterinarians who euthanize, and eight university personnel who harvest livestock were selected from across the United States (total *n* = 28). Participants were selected for their varying involvement in husbandry tasks. Animal shelter employees see and interact with individual animals daily, veterinarians see individual animals a few times per year but see them for many years, and university personnel who harvest livestock typically do not have any involvement with the animals outside of harvest. See Table 1 for a breakdown of demographic information. Missing demographic information was due to omissions by the participants. Seventeen men and eleven women participated in Experiment 1. Participants were contacted through their place of work and asked if they would be willing to participate in a study on occupational stress. All animal shelter employees and veterinarians were working in Texas, United States. Due to limited availability of universities that harvest livestock, university personnel were recruited from three states within the United States.

#### 2.1.2. General Procedure

**Figure 1:**
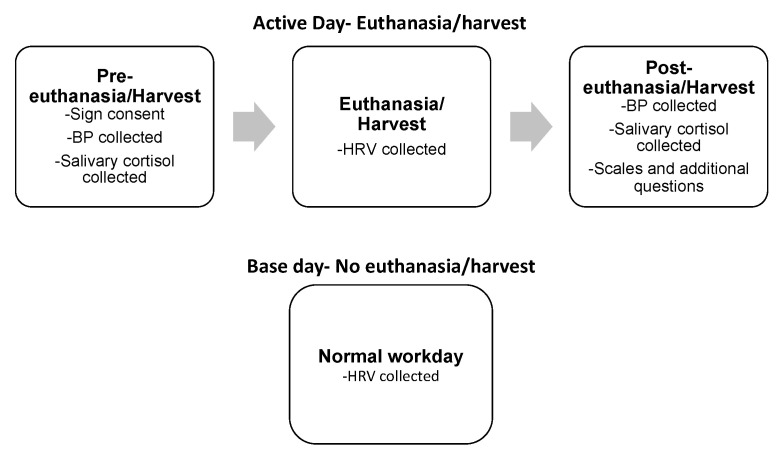
Shows a visual representation of methods for Experiment 1. BP: blood pressure; HRV: heart rate variability.

Figure 1 is a visual representation of the procedure for Experiment 1. Participants were given the Polar H7 (Polar Polar Electro Oy, Kempele, Finland) band to put around their chest. Once the band was in place, the researcher would check the connection with the V800 watch and then start data collection. Next, blood pressure and a saliva sample were taken. Then the euthanasia/harvesting began. Immediately following the euthanasia/harvest, the V800 watch was turned off and blood pressure and a saliva sample were collected again. If time allowed, the participant then completed the surveys and answered the additional questionnaire. Researchers attempted to make data collection as unobtrusive as possible, so if necessary, the participant was able to answer the surveys and questions later in their shift.

#### 2.1.3. Physiological Measures

Heart rate variability (HRV) was collected throughout euthanasia/harvest using Polar V800 and H7. Participants were instructed to get the sensors of the H7 band damp using water from the sink and then strap the band around their chest, with the sensor positioned just below their sternum. The author (AA) confirmed that there was skin-to-sensor contact and that the band was positioned properly. Correct location was confirmed by initiating the R-R testing function (interval between heart beats) on the polar watch. Once the V800 watch successfully connected with the H7 band, HRV data collection began. The duration of the euthanasia/harvest varied by participant, depending on the number of animals killed (range: 26 min to 8.6 h, mean: 2.6 h). There are a variety of time domain HRV calculations. SDNN, or the standard deviation of NN intervals, is the most common way to analyze HRV in 24 h frames [44]. NN intervals are the interbeat intervals from which artifacts have been removed [45]. However, it is not good for short-term (~5 min intervals) evaluation [45]. rMSSD, or the root-mean-square of successive differences of NN intervals, and pNN50, or the percentage of adjacent NN intervals that differ from each other by more than 50 ms, are common short-term HRV measures [45] and were therefore used for data analysis in this study. Both rMSSD and pNN50 are also controlled by the parasympathetic nervous system [45,46].

On a day when participants were not actively euthanizing or harvesting, they wore the heart rate monitor for the same amount of time as they did for the euthanasia/harvest. Due to the varying durations for euthanasia/harvest, the duration of the control period varied equivalently.

Blood Pressure (BP) was measured by a trained researcher (AA) pre- and post- euthanasia/harvest using an ADC 760 sphygmomanometer (American Diagnostic Corporation, Hauppauge, NY, USA) and 3M Littmann 5870 Classic III stethoscope (Littmann, St. Paul, MN, USA). The researcher followed the BP measuring protocol described in the Guyton and Hall Textbook of Medical Terminology [47]. Due to organizational policies and the sensitive nature of the work, the researcher (AA) was not always present for the euthanasia/slaughter. Therefore, the time between killing and the post-blood pressure reading was variable.

Saliva samples were collected pre- and post-euthanasia/slaughter using the Salimetrics Child Swabs (Salimetrics, State College, PA, USA) to be assayed for salivary cortisol. The Salimetrics protocol [48] was followed. Due to the variation in time of day of euthanasia/harvest, the change in salivary cortisol across jobs was dropped from the analysis [49]. Individual changes in salivary cortisol were included in the analysis.

#### 2.1.4. Psychometric Scales

Impact of Event Scale-Revised (IES-R) [50]: This 22-item scale is an indicator of Post-Traumatic Stress Disorder (PTSD). Respondents are asked to think about the past seven days, with respect to a traumatic experience, and indicate how much on a scale of 0 (not at all) to 4 (extremely) they have been distressed by certain difficulties. For Experiment 1, participants were asked to think about a particularly difficult euthanasia or harvest when answering the questions to focus on job-related trauma. Possible scores on this scale range from 0 to 88. A score of 24 or more indicates that PTSD is a clinical concern, and a score of 33 and above indicates the best probable cutoff for a diagnosis of PTSD [50,51]. The Cronbach’s alpha for this experiment was 0.94.

Professional Quality of Life Scale (ProQOL) [52]: This 30-item scale consists of three subscales: compassion satisfaction—the enjoyment one gains from helping others through their work, burnout—the feeling that one’s work is no longer making a difference, and secondary traumatic stress—trauma experienced through someone or something else [29]. Compassion fatigue has been referred to as the combination of burnout and secondary traumatic stress [29]. Each subscale consists of 10 questions which ask respondents to indicate how often they feel a certain way or exhibit certain behaviors on a scale from 1 (Never) to 5 (Very often). Scores on each of the subscales range from 10 to 50. The Cronbach’s alpha for compassion satisfaction, burnout, and secondary traumatic stress in this study was 0.84, 0.75, and 0.85, respectively.

#### 2.1.5. Additional Questions

Participants were asked the 11 questions regarding work and euthanasia/harvest history, marriage, spirituality, and pets at home by the researcher (AA).

### 2.2. Experiment 2

The purpose of Experiment 2 was to evaluate whether physically participating in killing is necessary to see a decline in occupational health in animal-care employees. Previous research suggested that moral injury was higher in animal shelter employees who euthanize, so the Moral Injury Event Scale was added to this study [3].

#### 2.2.1. Participants

A convenience sample of thirteen animal shelter employees who regularly euthanize, thirteen animal shelter employees who do not regularly euthanize, and fifteen pet hotel employees were selected (total *n* = 41). Participants were selected for their varying exposures to euthanasia. Euthanizing regularly was defined as at least once per week. Participants were contacted through their place of work. The thirteen animal shelter employees who euthanize regularly were recruited from Experiment 1. All animal shelter employees were currently working in Texas animal shelters. The pet hotel employees were currently working at a large pet hotel in Virginia. Experiment 2 included 20 men and 21 women. See Table 2 for a breakdown of the demographic information.

#### 2.2.2. Physiological Measures

Heart rate variability was collected during an entire workday using Polar V800 and H7. Animal shelter employees who euthanize were recorded on a day when they were not euthanizing to reflect the other jobs’ lack of euthanasia. Average shift duration was 8 h, but ranged from 5 to 12 h. The V800 and H7 were fit and synched using the same method as Experiment 1. Blood Pressure was measured at the beginning of the participant’s shift using the same method as in Experiment 1.

Cortisol Awakening Response was measured using saliva samples collected first thing in the morning using the Salimetrics Child Swabs (Salimetrics) [53]. Cortisol awakening response is a reliable indicator of an individual’s adrenocortical activity and is subject to less variance than salivary cortisol sampled later in the day [54]. The night before, participants were sent home with a swab, a vial, an icepack, and an insulated envelope. They were instructed to follow the Salimetrics protocol [48]. They were then told to put the swab in the tube and place the tube, along with the activated icepack, in the envelope and bring the entire package to work. The first author (AA) then met the participant at work and collected the sample.

#### 2.2.3. Psychometric Scales

The Impact of Event Scale-Revised (IES-R) and Professional Quality of Life Scale (ProQOL) were administered. Participants were instructed to think about their current job when answering the questions. The Cronbach’s alpha for IES-R for Experiment 2 was 0.94. The Cronbach’s alpha for compassion satisfaction, burnout, and secondary traumatic stress for Experiment 2 were 0.87, 0.71, and 0.85, respectively.

The Moral Injury Event Scale (MIES)**,** originally formulated for military personnel in 2013, assesses the impact of moral stress in both personal offenses and perceived betrayals [55]. A higher score on the MIES indicates lower moral injury. This scale has also been used in animal shelter employees to detect moral injury [3]. Questions 7–9 were adapted to reflect animal shelter and pet hotel employees. Question 7 originally read “I feel betrayed by leaders who I once trusted” and was changed to “I feel betrayed by superiors who I once trusted” for both animal shelter and pet hotel employees. Question 8 originally read “I feel betrayed by fellow service members who I once trusted” and was changed to “I feel betrayed by fellow employees who I once trusted” for both animal shelter and pet hotel employees. Question 9 originally read “I feel betrayed by others outside the U.S. military who I once trusted” and was changed to “I feel betrayed by others outside the animal shelter who I once trusted” and “I feel betrayed by others outside the pet hotel who I once trusted” for animal shelter and pet hotel employees, respectively. The Cronbach’s alpha for the nine-item, adapted, scale in this study was 0.86. Participants were instructed to think about their job at animal services / pet hotel when answering the scale.

#### 2.2.4. Additional Questions

Methods were the same as in Experiment 1.

#### 2.2.5. Data Analysis

Table 3 presents the averages of the psychometric measures across jobs for Experiments 1 and 2. Analyses were performed using R (R Core Team, Vienna, Austria, 2016). HRV data were analyzed using RHRV [56]. A principle component analysis (PCA) was run on the physiological measures in both Experiments 1 and 2. Due to the small amount of variance explained by the components in Experiment 1, each variable was analyzed separately. In Experiment 2, 71% of the variance was explained by two components. PC1 showed highest loadings (loadings > 0.3) for heart rate variability measures: rMSSD and pNN50, heart rate, and waking salivary cortisol. PC2 showed highest loadings for systolic and diastolic blood pressure. The premise of Experiment 1 was to test the impact of providing husbandry on occupational stress; therefore, contact with animals was used as the main independent variable. Linear regression models were run on each of the dependent variables by contact with animals, controlling for gender, age, smoking, marriage, spirituality, pet ownership, job, months on the job, total euthanasia, average number of euthanasias per session, and frequency of euthanasia. Each model was reduced through nest model comparison using the F test in R [57]. The variables remaining in the model are reported in Table 4. Paired *t*-tests were run on pre- versus post-euthanasia for blood pressure, heart rate, and saliva as well as on base versus active average heart rate, pNN50, rMSSD. In Experiment 2, linear regression models were run on each of the dependent variables by job, controlling for gender, age, smoking, marriage, spirituality, pet ownership, job, contact with animals, and months on the job. Each model was reduced similarly to Experiment 1. The variables retained variables are reported in Table 5.

## 3. Results

### 3.1. Experiment 1

**Figure 2:**
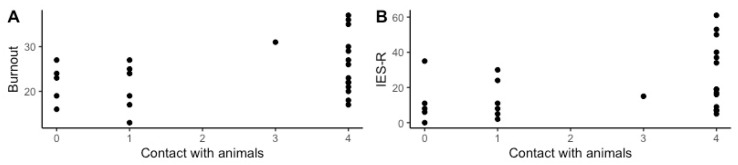
The significant differences in Burnout (**A**) and IES-R (**B**) by contact with animals. Possible burnout scores ranged from 10 to 50 and possible IES-R scores ranged from 0 to 88. Greater contact with animals predicted lower burnout (F (1) = 12.408, *p* = 0.003) and higher IES-R scores (F (1) = 10.012, *p* = 0.005). IES-R: Impact of Event Scale-Revised.

**Figure 3:**
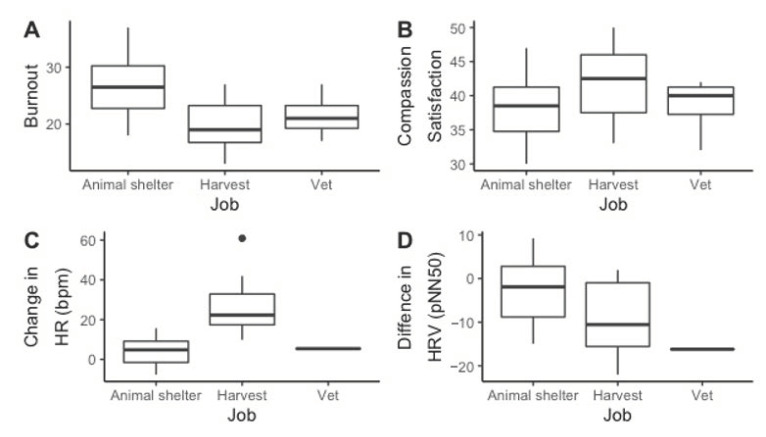
The significant differences in Burnout (**A**), Compassion Satisfaction (**B**), Change in HR (**C**), and Difference in HRV (**D**) by job. Possible burnout and compassion satisfaction scores range from 10 to 50 points. Animal shelter employees had significantly higher burnout (F (2) = 7.246, *p* = 0.004), lower compassion satisfaction (F (2) = 3.918, *p* = 0.035), and less change in HR (F (2) = 12.535, *p* < 0.001), and less difference in HRV (F (2) = 3.842, *p* = 0.042). HR: heart rate.

Table 4 reports the output from the reduced linear models. See Table S1 for the output from all the original and reduced linear models. In the reduced linear model predicting burnout, higher burnout was significantly associated with less contact with animals (F (1) = 12.408, *p* = 0.003), male gender (F (1) = 6.657, *p* = 0.019), not being married (F (1) = 7.093 *p* = 0.016), not being spiritual (F (1) = 24.884, *p* < 0.001), having pets at home (F (1) = 7.246, *p* = 0.015), and working in an animal shelter (F (2) = 7.246, *p* = 0.004). See Figure 2A and Figure 3A for a visual representation of the differences in burnout across job and contact with animals, respectively. In the reduced linear model predicting compassion satisfaction, not working as an animal shelter employee (F (2) = 3.918, *p* = 0.035) and owning a pet (F (1) = 8.107, *p* = 0.009) were statistically significantly associated with higher compassion satisfaction. See Figure 3B for a visual representation of the differences in compassion satisfaction across jobs. In the reduced linear model predicting IES-R scores, greater contact with animals (F (1) = 10.012, *p* = 0.005), not being married (F (1) = 6.081 *p* = 0.024), months on the job (F (1) = 12.816, *p* = 0.002), and greater average number of euthanasias per session (F (1) = 13.315, *p* = 0.002) were statistically significantly associated with higher IES-R scores (more likely to have PTSD). See Figure 2B for a visual representation of the differences in IES-R across contact with animals. In the reduced linear model predicting the difference in pNN50 between active euthanasia and work with euthanasia, working in an animal shelter (F (2) = 3.842, *p* = 0.042) and not owning a pet (F (1) = 10.555, *p* = 0.005) were statistically significantly associated with less decrease in pNN50. See Figure 3D for a visual representation of the differences in change in pNN50 across jobs. In the reduced linear model predicting secondary traumatic stress, greater total slaughter/euthanasias performed (F (1) = 6.373, *p* = 0.019) was statistically significantly associated with higher secondary traumatic stress. In the reduced linear model predicting the change in HR, not working in an animal shelter (F (2) = 12.535, *p* < 0.001) and owning a pet (F (1) = 5.489, *p* = 0.0312) were statistically significantly associated with a greater change in HR. See Figure 3C for a visual representation of the differences in change in HR across jobs. In the reduced linear model predicting the change in diastolic blood pressure, not owning a pet (F (1) = 5.433, *p* = 0.029) was statistically significantly associated with greater change in diastolic blood pressure. In the reduced linear model predicting change in systolic blood pressure, being married (F (1) = 8.542, *p* = 0.008) and greater average number of euthanasias per session (F (1) = 6.919, *p* = 0.016) were statistically significantly associated with greater change in systolic blood pressure. There was no statistically significant association in the reduced model predicting the difference in rMSSD.

**Figure 4:**
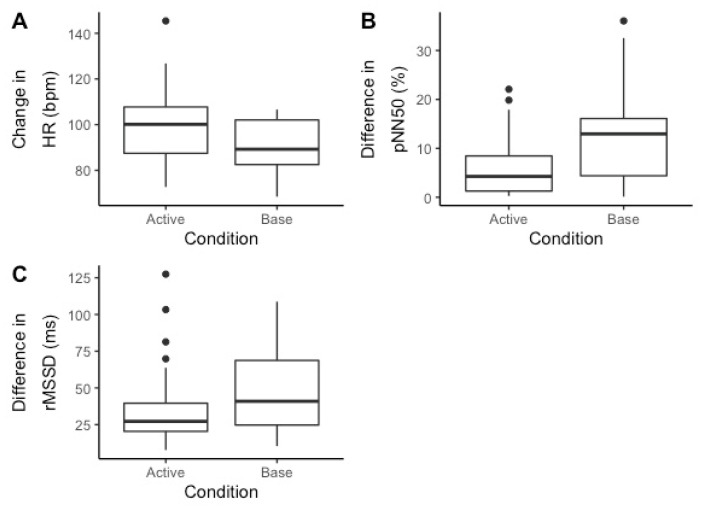
The difference in HR (**A**) and HRV (**B**,**C**) measures during Active (euthanizing) vs. base (non-euthanizing) days. Average heart rate (t (20) = 3.785, *p* = 0.001) was statistically significantly higher and pNN50 (t (20) = −3.171, *p* = 0.005) was lower during killing. There were no significant differences in rMSSD.

Systolic blood pressure (t (25) = −2.309, *p* = 0.029) was statistically significantly higher following euthanasia. Salivary cortisol (t (25) = 2.826, *p* = 0.009) was statistically significantly higher prior to euthanasia. There were no significant differences in heart rate or diastolic blood pressure. Average heart rate (t (20) = 3.785, *p* = 0.001) was statistically significantly higher and pNN50 (t (20) = −3.171, *p* = 0.005) was lower during killing. There were no significant differences in rMSSD. See Figure 4 for a visual representation.

### 3.2. Experiment 2

Table 5 reports the output from all the linear models. Compassion satisfaction was the only dependent variable that remained significant following the model reduction. See Table S2 for the output from all the original and reduced linear models. In the reduced linear model predicting compassion satisfaction, female gender (F (1) = 9.701, *p* = 0.005), being married (F (1) = 5.553, *p* = 0.027), and not owning a pet (F (1) = 7.416, *p* = 0.012) were statistically significantly associated with a higher score. Contrary to our hypothesis, there were no statistically significant associations in the reduced models predicting burnout, moral injury, IES-R scores, secondary traumatic stress, PC1, and PC2 by job.

## 4. Discussion

### 4.1. Experiment 1

A greater change in HR and less of a decrease in HRV (pNN50) was significantly related to not working in an animal shelter. HR was significantly higher and pNN50 was significantly lower on killing days compared to a non-killing days across jobs. Increased heart rate is associated with decreased HRV and indicates arousal, and both are often associated with emotional stress [58,59,60,61]. This suggests that killing, no matter the contact with animals prior, may be emotionally stressful. Interestingly, HRV hyporeactivity has been used as an indicator of both depression [62] and PTSD [63]. HRV biofeedback training has been successfully implemented as a treatment for individuals with anxiety, depression, and PTSD [64,65,66]. A study using biofeedback training as a predeployment protocol found that participants with the biofeedback training experienced less arousal during postdeployment combat situations [67]. This indicates that it may be beneficial for animal-care organizations that engage in killing to incorporate biofeedback training into protocols to decrease the arousal associated with killing.

The statistically significant decrease in salivary cortisol levels between pre- and post-euthanasia/slaughter can be explained by the diurnal nature of salivary cortisol levels [49]. It should be noted that time of day was not controlled for; all harvesting began prior to 9:00 a.m., whereas the majority of animal shelter and veterinary euthanasia occurred in the afternoon and evening. Cortisol spikes approximately 30 min after awakening and then continues to decline throughout the day, with the biggest decline occurring in the morning [54,68]. It continues to decrease throughout the day, so a plateau or slight decrease may indicate stress. The duration of killing was also not controlled for; therefore, the difference in pre- and post-salivary cortisol could not be compared across jobs.

Previous research has shown that BP increases during acute stress; however, we only saw an increase in systolic pressure following killing [69]. An increase in systolic reactivity during a stressor is commonly associated with hypertension [70]. Occupational as well as psychosocial stress have been correlated with hypertension [71,72]. We did not ask participants about comorbid disorders, so this suggestion of hypertension is speculative. One potential explanation for the inconsistency and lack of increase in diastolic BP is that BP was taken as close to the cessation of killing as possible, but it was not always feasible. Due to the sensitive nature of euthanasia and harvest, the author (AA) was not always able to be present during the actual process and did not know the time between last killing and the post BP measurement. It is possible that BP did increase during killing, but had returned to baseline before the author had the opportunity to measure this change.

Increased contact with animals predicted higher IES-R scores. More than one third (*n* = 6) of animal shelter employees scored at or above a 33, which represents the cutoff for a probable diagnosis of PTSD [51]. This is higher than previous research in shelter employees, which found that approximately one-fifth of the population scored high enough for a probable diagnosis of PTSD [3]. It is estimated that 3.5% of the population is suffering from PTSD in a 12 month period [73]. Data from this study suggest that animal shelter employees may be more likely than the general population to have PTSD in a given year. While the average number of animals killed in each session did statistically significantly influence the model predicting IES-R scores, neither frequency nor total number of animals killed statistically significantly predicted IES-R scores. This supports recent research in laboratory animal personnel [17] and animal-care professionals [18], which found that other job stressors contribute more to occupational stress than euthanasia.

Decreased contact with animals and job predicted burnout, but not secondary traumatic stress. While burnout was statistically significantly different across jobs, no participants scored a 42 or above, indicating high burnout. There were also no participants who scored in the high range of secondary traumatic stress. The majority of participants scored in the average range for both burnout (*n* = 16) and secondary traumatic stress (*n* = 11), suggesting that both are still a concern. This supports previous studies that also found very low rates of high employee burnout and secondary traumatic stress, but high rates of average burnout in animal-care employees [3,19].

Contact with animals did not statistically significantly predict compassion satisfaction; however, marriage, job, and pet ownership were statistically significant in this model. This aligns with previous research that support from significant others [74] predicts compassion satisfaction. Lower compassion satisfaction scores were associated with working in an animal shelter. A score of 23–41 indicates average compassion satisfaction, and 42 or more indicates very high compassion satisfaction [52]. All participants had average-to-high compassion satisfaction, indicating that they all felt like they were making a difference through their work. The average-to-high compassion satisfaction scores are similar to those found in previous studies, which found average compassion satisfaction scores between 39 and 42 in animal shelter employees [3,18].

### 4.2. Experiment 2

There were no statistically significant differences in the reduced models predicting burnout, moral injury, secondary traumatic stress, PC1 and PC2 between animal shelter employees who euthanize regularly, those who do not euthanize regularly, and people who work in a pet hotel. This contradicts the hypothesis that the act of killing is necessary to develop compassion fatigue and moral injury. It also further supports the findings from Monaghan and colleagues (2020) that non-euthanasia related job demands are better predictors of burnout and secondary traumatic stress than euthanasia. In the context of the results from Experiment 1, these data suggest that simply providing husbandry and constant care for animals may be enough to develop compassion fatigue. This aligns with research that has found increased prevalence of compassion fatigue in a variety of human care-giving careers in which death is not a typical outcome [75,76]. The lack of relationship between euthanasia and moral injury contradicts previous research which found moral injury to be higher in individuals who do euthanize [3]. The average moral injury score across all jobs was 37, which is similar to previously reported data that found the median scores of 35 and 44 in shelter employees who euthanize and who do not, respectively. The current data suggest that moral injury may be a concern for many animal-care professionals.

### 4.3. Limitations and Future Directions

This study was exploratory as physiological measures of compassion fatigue and moral injury in animal-care employees had never been previously assessed. The recruitment of participants proved to be a great challenge due to the sensitive nature of the subject matter, which led to a small sample size and incomplete data sets for the majority of the veterinarians, limiting interpretation of the data. All animal shelter and veterinary participants were from Texas, pet hotel employees were all from Virginia, and university personnel who harvest were from Texas, Oklahoma, and Virginia. There is a possibility that the geographic differences impacted the physiologic and psychometric measures. Comorbid disorders and/or previous experience may have also impacted the physiologic and psychometric measures.

The lack of control for time of day and length of killing in Experiment 1 limited the comparisons that could be done. Due to the diurnal nature of salivary cortisol, and the variation of time of day of killing, salivary cortisol could not be compared across jobs. Practically, it was not possible to control for this as shelters, veterinarians, and universities have set times when they euthanize and harvest.

The time constraints associated with each job also led to some participants answering the survey questions following slaughter or euthanasia, while others answered the questions on another day. It is possible that the time in between killing and answering the psychometric measures may have had an impact on scores.

## 5. Conclusions

The overall aim of these studies was to evaluate the physiological and psychometric correlates of caring and killing in animal-care work. Experiment 1 found that increased contact with animals prior to killing predicted higher burnout and traumatic stress and that killing caused an increase in HR and a decrease in HRV. Experiment 2 found that jobs with greater euthanasia frequency did not predict any of the psychometric or physiological measures, suggesting that killing may not be the biggest unique predictor of occupational stress. Future research should focus on non-euthanasia-related job stressors as well as interventions to combat those stressors in animal-care staff.

## Figures and Tables

**Table 1:** Shows the demographic information for Experiment 1.

	Animal Shelter	Veterinarian	University Employee Who Harvests
Gender			
Male	9	2	6
Female	7	2	2
No response	0	0	0
Age			
<20	0	0	0
20–40	10	2	8
40–60	6	1	0
60+	0	1	0
No response	0	0	0
Smoke			
Yes	4	0	0
No	12	2	8
No response	0	2	0
Spiritual			
Yes	14	4	8
No	2	0	0
No response	0	0	0
Married			
Yes	8	4	1
No	8	0	7
No response	0	0	0
Pets at home			
Yes	15	3	5
No	1	0	3
No response	0	1	0
Contact with animals			
None	0	0	5
Not much	3	0	3
Some	0	0	0
Moderate	1	0	0
A lot	12	2	0
No response	0	2	0
Months on the job			
Range:	3–312	12–60	12–216
Mean:	96.81	36.00	70.50
Median:	84	36	42
No response	0	2	0
Total euthanized/harvested:			
<100	0	0	3
100–1000	4	2	3
1001–10,000	6	0	2
10,001–100,000	3	0	0
>100,000	1	0	0
No response	2	2	0
Frequency of euthanasia/harvest:			
Never	0	0	0
<1/month	0	0	2
1/month	0	0	1
2/month	0	1	2
1/week	2	0	3
2/week	1	0	0
>3/week	4	0	0
Daily	8	0	0
No response	1	3	0
Euthanized/harvested at a time:			
Range:	5–90	1	3–13
Mean:	21.13	1.00	6.50
Median:	15.5	1	6.5
No response	0	3	0

**Table 2:** The demographic information for Experiment 2.

	Animal Shelter–Regular Euthanasia	Animal Shelter–Irregular or No Euthanasia	Pet Hotel
Gender			
Male	6	6	8
Female	7	7	7
No response	0	0	0
Age			
<20	0	1	4
20–40	7	5	10
40–60	6	4	0
60+	0	3	1
No response	0	0	0
Smoke			
Yes	3	1	7
No	10	11	7
No response	0	1	1
Spiritual			
Yes	12	11	10
No	1	0	4
No response	0	2	1
Married			
Yes	6	6	1
No	7	5	13
No response	0	2	1
Pets at home			
Yes	13	11	10
No	0	1	3
No response	0	1	2
Contact with animals			
None	0	0	0
Not much	3	5	0
Some	0	1	0
Moderate	1	1	0
A lot	9	5	15
No response	0	0	1
Months on the job			
Range:	3–312	0.5–132	6–132
Mean:	97.96	65.08	39.08
Median:	84	48	24
No response	0	0	1

**Table 3:** The averages of the psychometric measures across jobs for Experiments 1 and 2.

	Impact of Event Scale Scores	Compassion Satisfaction	Burnout	Secondary Traumatic Stress	Moral Injury *
Experiment 1					
Animal shelter					
Mean	26.88	37.94	27.06	23.75	
SE	4.36	1.23	1.40	1.73	
Range	5:61	30:47	18:37	13:38	
University employee who harvests livestock					
Mean	9.38	42.00	19.75	17.25	
SE	3.86	2.15	1.62	1.63	
Range	0:35	33:50	13:27	13:27	
Veterinarian					
Mean	11.00	38.50	21.50	22.50	
SE	4.60	2.25	2.10	1.32	
Range	0:20	32:42	17:27	20:26	
Experiment 2					
Pet hotel					
Mean	18.14	38.07	23.00	20.50	35.86
SE	3.60	2.12	1.48	1.11	2.68
Range	0:46	25:50	10:32	16:29	18:50
Animal shelter: irregular euthanasia					
Mean	21.54	39.85	23.54	22.69	36.69
SE	5.39	1.62	1.60	2.15	2.63
Range	0:56	31:50	13:34	10:37	20:54
Animal shelter: regular euthanasia					
Mean	23.69	37.77	27.00	23.15	38.77
SE	4.49	1.26	1.36	1.95	3.10
Range	5:61	30:47	21:36	13:38	22:54

* Note. Higher moral injury scores indicate lower moral injury.

**Table 4:** Shows the reduced linear model output for Experiment 1.

Variable	Df	Sum Sq	Mean Sq	Estimate	Std. Error	F Value	Pr(>F)	Significance
**Burnout–reduced model**								
Contact with animals	1	130.72	130.72	−1.70	0.75	12.41	0.00	*
Gender	1	70.13	70.13	−3.92	1.54	6.66	0.02	*
Age	1	24.03	24.03	5.34	2.06	2.28	0.15	
Married	1	74.73	74.73	−8.741	1.98	7.09	0.02	***
Spiritual	1	262.16	262.16	−9.20	2.90	24.88	0.00	**
Job	2	162.18	81.09	−15.22	3.27	7.70	0.00	***
Pets	1	76.34	76.34	−6.25	2.32	7.25	0.02	**
Residuals	17	179.1	10.54	NA	NA	NA	NA	
**Compassion satisfaction–reduced model**								
Married	1	28.02	28.02	5.76	2.03	1.54	0.23	
Job	2	143.05	71.52	8.60	2.26	3.92	0.04	*
Pets	1	148.00	148.00	7.61	2.67	8.11	0.01	**
Residuals	22	401.61	18.26	NA	NA	NA	NA	
**Secondary traumatic stress–reduced model**								
Total euthanasias/harvest performed	1	183.69	183.69	2.78	1.10	6.37	0.02	*
Residuals	22	634.14	28.83	NA	NA	NA	NA	
**Impact of event scale score–reduced model**								
Contact with animals	1	1164.74	1164.74	6.00	1.53	10.01	0.01	**
Age	1	21.28	21.28	−17.72	8.10	0.18	0.67	
Smoke	1	73.43	73.43	17.10	7.47	0.63	0.44	
Married	1	707.38	707.38	−19.21	5.51	6.08	0.02	*
Spiritual	1	123.19	123.19	27.66	12.11	1.06	0.32	
Months on the job	1	1490.95	1490.95	0.14	0.04	12.82	0.00	**
Average number of euthanasias per session	1	1548.92	1548.92	0.65	0.18	13.32	0.00	**
Residuals	18	2093.99	116.33	NA	NA	NA	NA	
**Change in heart rate–reduced model**								
Job	2	2716.61	1358.30	27.76	5.09	12.54	0.00	***
Pets	1	594.83	594.83	14.60	6.23	5.49	0.03	*
Residuals	17	1842.20	108.37	NA	NA	NA	NA	
**Change in systolic pressure–reduced model**								
Married	1	303.60	303.60	6.06	2.51	8.54	0.01	**
Average number of euthanasias per session	1	245.93	245.93	0.18	0.07	6.92	0.02	*
Residuals	21	746.43	35.54	NA	NA	NA	NA	
**Change in diastolic pressure–reduced model**								
Pets	1	330.98	330.98	−11.20	4.80	5.43	0.03	*
Residuals	23	1401.26	60.92	NA	NA	NA	NA	
**Difference in pNN50–reduced model**								
Job	2	322.17	161.09	−10.31	3.16	3.84	0.04	**
Pets	1	442.55	442.55	−12.59	3.88	10.56	0.01	**
Residuals	17	712.77	41.93	NA	NA	NA	NA	

Note: * *p* < 0.05, ** *p* < 0.01, *** *p* < 0.001.

**Table 5:** Shows the reduced linear model output for Experiment 2.

Variable	Df	Sum Sq	Mean Sq	Estimate	Std. Error	F Value	Pr(>F)	Significance
**Compassion satisfaction–reduced model**								
Gender	1	214.520	214.52	5.28	1.75	9.70	0.01	**
Married	1	122.800	122.80	4.72	1.89	5.55	0.03	*
Pets	1	163.990	163.99	−6.91	2.54	7.42	0.01	*
Residuals	25	552.820	22.11	NA	NA	NA	NA	

Note: * *p* < 0.05, ** *p* < 0.01.

## Supplementary Materials

The following are available online at https://www.mdpi.com/1660-4601/17/24/9196/s1, Table S1: Experiment 1 Analysis, Table S2: Experiment 2 Analysis.
