# Supplementary material for: The Impact of Caring and Killing on Physiological and Psychometric Measures of Stress in Animal Shelter Employees: A Pilot Study
Source: Int J Environ Res Public Health. 2020 Dec 9;17(24):9196. doi: 10.3390/ijerph17249196 (PMC7764342; doi:10.3390/ijerph17249196)
Supplement: Supplementary file 1 [file ijerph-17-09196-s001.pdf]

Table S1. Experiment 1 Analysis.

Variable	Df	Sum Sq	Mean Sq	Estimate	Std. Error	F value	Pr(>F)	Significance
**Burnout**								
Original Model								
Contact with animals	1	114.679	114.679	−1.591	2.131	8.962	0.017	*
Gender	1	76.902	76.902	−4.468	2.419	6.009	0.040	*
Age	1	0.174	0.174	1.603	4.474	0.014	0.910	
Smoke	1	38.785	38.785	3.225	3.636	3.031	0.120	
Married	1	57.927	57.927	−8.404	3.309	4.527	0.066	
Spiritual	1	192.529	192.529	−7.875	6.273	15.045	0.005	**
Job	2	185.510	92.755	−12.801	8.876	7.248	0.016	*
Pets	1	57.422	57.422	−4.012	5.243	4.487	0.067	
Months on the job	1	0.311	0.311	0.008	0.031	0.024	0.880	
Total euthanasias/ harvest performed	1	0.009	0.009	−0.113	2.049	0.001	0.980	
Average number of euthanasias per session	1	1.465	1.465	0.014	0.110	0.114	0.744	
Frequency of euthanasia	1	1.186	1.186	0.469	1.541	0.093	0.769	
Residuals	8	102.374	12.797	NA	NA	NA	NA	
Reduced Model								
Contact with animals	1	130.719	130.719	−1.695	0.745	12.408	0.003	*
Gender	1	70.131	70.131	−3.917	1.537	6.657	0.019	*
Age	1	24.029	24.029	5.335	2.064	2.281	0.149	
Married	1	74.731	74.731	−8.741	1.975	7.093	0.016	***
Spiritual	1	262.158	262.158	−9.198	2.899	24.884	0.000	**
Job	2	162.179	81.089	−15.215	3.273	7.697	0.004	***
Pets	1	76.340	76.340	−6.253	2.323	7.246	0.015	**
Residuals	17	179.099	10.535	NA	NA	NA	NA	
**Compassion satisfaction**								
Original Model								
Contact with animals	1	63.789	63.789	0.512	2.564	3.443	0.101	
Gender	1	3.582	3.582	2.402	2.911	0.193	0.672	
Age	1	0.006	0.006	−6.509	5.383	0.000	0.986	
Smoke	1	0.363	0.363	0.071	4.375	0.020	0.892	
Married	1	122.948	122.948	13.397	3.982	6.636	0.033	*
Spiritual	1	121.957	121.957	4.053	7.548	6.583	0.033	*
Job	2	66.407	33.203	5.459	10.680	1.792	0.227	
Pets	1	156.463	156.463	5.202	6.308	8.445	0.020	*
Months on the job	1	1.486	1.486	−0.008	0.038	0.080	0.784	

Total euthanasias/ harvest performed	1	3.722	3.722	−1.058	2.465	0.201	0.666	
Average number of euthanasias per session	1	15.652	15.652	−0.069	0.133	0.845	0.385	
Frequency of euthanasia	1	4.500	4.500	−0.914	1.854	0.243	0.635	
Residuals	8	148.215	18.527	NA	NA	NA	NA	
Reduced Model								
Married	1	28.017	28.017	5.757	2.031	1.535	0.228	
Job	2	143.046	71.523	8.601	2.256	3.918	0.035	*
Pets	1	147.996	147.996	7.614	2.674	8.107	0.009	**
Residuals	22	401.608	18.255	NA	NA	NA	NA	
**Secondary traumatic stress**								
Original Model								
Contact with animals	1	30.683	30.683	1.246	2.927	1.272	0.292	
Gender	1	0.031	0.031	0.710	3.322	0.001	0.972	
Age	1	6.660	6.660	−6.661	6.143	0.276	0.614	
Smoke	1	102.531	102.531	7.311	4.993	4.249	0.073	
Married	1	0.601	0.601	−7.520	4.544	0.025	0.879	
Spiritual	1	88.693	88.693	3.229	8.614	3.676	0.092	
Job	2	162.290	81.145	−5.354	12.189	3.363	0.087	
Pets	1	86.528	86.528	−0.022	7.199	3.586	0.095	
Months on the job	1	21.284	21.284	0.054	0.043	0.882	0.375	
Total euthanasias/ harvest performed	1	9.188	9.188	3.067	2.813	0.381	0.554	
Average number of euthanasias per session	1	6.164	6.164	0.133	0.151	0.255	0.627	
Frequency of euthanasia	1	17.637	17.637	−1.809	2.116	0.731	0.417	
Residuals	8	193.028	24.129	NA	NA	NA	NA	
Reduced Model								
Total euthanasias/ harvest performed	1	183.690	183.693	2.776	1.100	6.373	0.019	*
Residuals	22	634.140	28.825	NA	NA	NA	NA	
**Impact of event scale score**								
Original Model								
Contact with animals	1	428.522	428.522	2.369	4.892	6.358	0.036	*
Gender	1	99.132	99.132	−8.261	5.552	1.471	0.260	
Age	1	218.139	218.139	−25.309	10.268	3.236	0.110	
Smoke	1	387.435	387.435	11.112	8.345	5.748	0.043	*
Married	1	9.118	9.118	−9.984	7.594	0.135	0.723	
Spiritual	1	176.583	176.583	6.794	14.397	2.620	0.144	

Job	2	744.446	372.223	19.628	20.372	5.522	0.031	*
Pets	1	335.239	335.239	14.794	12.032	4.974	0.056	
Months on the job	1	3.637	3.637	0.125	0.072	0.054	0.822	
Total euthanasias/ harvest performed	1	5.533	5.533	0.437	4.702	0.082	0.782	
Average number of euthanasias per session	1	828.204	828.204	0.517	0.253	12.287	0.008	**
Frequency of euthanasia	1	214.782	214.782	6.313	3.537	3.186	0.112	
Residuals	8	539.231	67.404	NA	NA	NA	NA	
Reduced Model								
Contact with animals	1	1164.735	1164.735	5.999	1.532	10.012	0.005	**
Age	1	21.278	21.278	−17.723	8.098	0.183	0.674	
Smoke	1	73.433	73.433	17.102	7.468	0.631	0.437	
Married	1	707.383	707.383	−19.205	5.508	6.081	0.024	*
Spiritual	1	123.191	123.191	27.664	12.113	1.059	0.317	
Months on the job	1	1490.954	1490.954	0.144	0.041	12.816	0.002	**
Average number of euthanasias per session	1	1548.920	1548.920	0.652	0.179	13.315	0.002	**
Residuals	18	2093.989	116.333	NA	NA	NA	NA	
Change in heart rate								
Original Model								
Contact with animals	1	1555.536	1555.536	6.390	12.733	5.032	0.075	
Gender	1	0.969	0.969	4.521	15.984	0.003	0.958	
Age	1	77.436	77.436	−14.772	35.050	0.251	0.638	
Smoke	1	283.818	283.818	−0.050	19.821	0.918	0.382	
Married	1	68.311	68.311	7.000	19.349	0.221	0.658	
Spiritual	1	215.444	215.444	14.865	34.788	0.697	0.442	
Job	2	672.107	336.053	44.365	52.308	1.087	0.405	
Pets	1	443.087	443.087	22.730	32.515	1.433	0.285	
Months on the job	1	13.501	13.501	0.060	0.210	0.044	0.843	
Total euthanasias/ harvest performed	1	0.185	0.185	1.319	10.790	0.001	0.981	
Average number of euthanasias per session	1	6.089	6.089	0.153	0.711	0.020	0.894	
Frequency of euthanasia	1	9.600	9.600	−1.436	8.148	0.031	0.867	
Residuals	5	1545.515	309.103	NA	NA	NA	NA	
Reduced Model								
Job	2	2716.606	1358.303	27.762	5.087	12.535	0.000	***
Pets	1	594.825	594.825	14.597	6.230	5.489	0.032	*

Residuals	17	1842.201	108.365	NA	NA	NA	NA	
Change in systolic pressure								
Original Model								
Contact with animals	1	132.901	132.901	7.704	4.271	3.754	0.101	
Gender	1	7.784	7.784	7.705	5.032	0.220	0.656	
Age	1	0.102	0.102	−7.194	9.293	0.003	0.959	
Smoke	1	89.108	89.108	4.126	6.070	2.517	0.164	
Married	1	127.681	127.681	7.315	6.087	3.606	0.106	
Spiritual	1	151.339	151.339	2.896	10.784	4.275	0.084	
Job	2	119.319	59.660	26.213	19.532	1.685	0.263	
Pets	1	61.868	61.868	15.989	9.132	1.748	0.234	
Months on the job	1	0.391	0.391	0.067	0.065	0.011	0.920	
Total euthanasias/ harvest performed	1	7.097	7.097	4.588	3.893	0.200	0.670	
Average number of euthanasias per session	1	85.275	85.275	0.382	0.196	2.409	0.172	
Frequency of euthanasia	1	49.665	49.665	−3.360	2.837	1.403	0.281	
Residuals	6	212.420	35.403	NA	NA	NA	NA	
Reduced Model								
Married	1	303.600	303.601	6.062	2.507	8.542	0.008	**
Average number of euthanasias per session	1	245.930	245.926	0.182	0.069	6.919	0.016	*
Residuals	21	746.430	35.544	NA	NA	NA	NA	
Change in diastolic pressure								
Original Model								
Contact with animals	1	17.415	17.415	−0.752	6.147	0.237	0.643	
Gender	1	213.168	213.168	−12.700	7.242	2.907	0.139	
Age	1	8.540	8.540	−4.570	13.375	0.116	0.745	
Smoke	1	40.743	40.743	0.478	8.736	0.556	0.484	
Married	1	13.107	13.107	9.894	8.760	0.179	0.687	
Spiritual	1	10.093	10.093	−17.019	15.520	0.138	0.723	
Job	2	287.730	143.865	6.262	28.110	1.962	0.221	
Pets	1	159.538	159.538	−19.278	13.142	2.176	0.191	
Months on the job	1	59.667	59.667	−0.023	0.094	0.814	0.402	
Total euthanasias/ harvest performed	1	4.398	4.398	−6.017	5.603	0.060	0.815	
Average number of euthanasias per session	1	204.220	204.220	−0.609	0.282	2.785	0.146	

Frequency of euthanasia	1	141.160	141.160	5.665	4.083	1.925	0.215	
Residuals	6	439.970	73.328	NA	NA	NA	NA	
Reduced Model								
Pets	1	330.980	330.980	−11.197	4.804	5.433	0.029	*
Residuals	23	1401.260	60.920	NA	NA	NA	NA	
Difference in rMSSD								
Original Model								
Contact with animals	1	36.137	36.137	−4.133	36.283	0.014	0.909	
Gender	1	2138.648	2138.648	−28.437	45.547	0.852	0.398	
Age	1	236.756	236.756	−32.685	99.878	0.094	0.771	
Smoke	1	2147.253	2147.253	68.695	56.483	0.855	0.397	
Married	1	1097.863	1097.863	−34.836	55.138	0.437	0.538	
Spiritual	1	155.213	155.213	−27.894	99.133	0.062	0.814	
Job	2	560.105	280.052	−1.064	149.057	0.112	0.897	
Pets	1	7753.342	7753.342	−48.045	92.654	3.089	0.139	
Months on the job	1	2643.868	2643.868	0.278	0.600	1.053	0.352	
Total euthanasias/ harvest performed	1	1666.723	1666.723	−33.525	30.748	0.664	0.452	
Average number of euthanasias per session	1	25.128	25.128	−0.986	2.025	0.010	0.924	
Frequency of euthanasia	1	2490.667	2490.667	23.129	23.219	0.992	0.365	
Residuals	5	12549.970	2509.994	NA	NA	NA	NA	
Reduced Model None								
Difference in pNN50								
Original Model								
Contact with animals	1	61.529	61.529	−1.703	4.281	1.761	0.242	
Gender	1	212.187	212.187	−6.683	5.374	6.072	0.057	
Age	1	34.481	34.481	2.855	11.785	0.987	0.366	
Smoke	1	273.384	273.384	11.710	6.665	7.823	0.038	*
Married	1	0.461	0.461	−3.153	6.506	0.013	0.913	
Spiritual	1	33.408	33.408	−10.302	11.697	0.956	0.373	
Job	2	73.016	36.508	−17.405	17.588	1.045	0.418	
Pets	1	341.989	341.989	−18.542	10.933	9.786	0.026	*
Months on the job	1	101.927	101.927	0.010	0.071	2.917	0.148	
Total euthanasias/ harvest performed	1	12.996	12.996	−3.606	3.628	0.372	0.569	
Average number of euthanasias per session	1	56.607	56.607	−0.305	0.239	1.620	0.259	

Frequency of euthanasia	1	5.769	5.769	1.113	2.740	0.165	0.701	
Residuals	5	174.730	34.946	NA	NA	NA	NA	
Reduced Model								
Job	2	322.173	161.087	−10.313	3.164	3.842	0.042	**
Pets	1	442.548	442.548	−12.591	3.875	10.555	0.005	**
Residuals	17	712.769	41.928	NA	NA	NA	NA	

Table S2. Experiment 2 Analysis.

Variable	Df	Sum Sq	Mean Sq	Estimate	Std. Error	F value	Pr(>F)	Significance
**Burnout**								
Original Model								
Job	2	75.886	37.943	0.926	4.498	1.422	0.267	
Gender	1	31.056	31.056	−0.640	2.331	1.164	0.295	
Age	1	1.517	1.517	0.951	1.729	0.057	0.814	
Smoke	1	0.262	0.262	1.445	2.601	0.010	0.922	
Married	1	70.247	70.247	−6.575	3.191	2.633	0.122	
Spiritual	1	3.414	3.414	0.129	2.856	0.128	0.725	
Contact with animals	1	0.032	0.032	0.120	1.286	0.001	0.973	
Pets	1	75.448	75.448	4.832	3.138	2.828	0.110	
Months on the job	1	40.894	40.894	0.033	0.027	1.533	0.232	
Residuals	18	480.210	26.678	NA	NA	NA	NA	
Reduced Model								
None								
**Moral injury**								
Original Model								
Job	2	44.537	22.269	2.827	8.626	0.227	0.799	
Gender	1	7.167	7.167	0.392	4.471	0.073	0.790	
Age	1	231.263	231.263	−4.351	3.316	2.357	0.142	
Smoke	1	14.777	14.777	0.749	4.987	0.151	0.703	
Married	1	115.978	115.978	6.715	6.121	1.182	0.291	
Spiritual	1	307.760	307.760	−8.774	5.478	3.136	0.093	
Contact with animals	1	184.867	184.867	2.988	2.466	1.884	0.187	
Pets	1	19.356	19.356	−2.403	6.018	0.197	0.662	
Months on the job	1	15.427	15.427	−0.020	0.051	0.157	0.696	
Residuals	18	1766.316	98.129	NA	NA	NA	NA	
Reduced Model								
None								
**Impact of event scale score**								
Original Model								
Job	2	458.846	229.423	0.774	13.261	0.989	0.391	
Gender	1	89.665	89.665	6.266	6.873	0.387	0.542	
Age	1	31.640	31.640	3.540	5.099	0.136	0.716	

Smoke	1	148.336	148.336	0.644	7.668	0.640	0.434	
Married	1	1180.079	1180.079	−22.497	9.410	5.088	0.037	*
Spiritual	1	200.930	200.930	5.835	8.422	0.866	0.364	
Contact with animals	1	238.142	238.142	−2.960	3.791	1.027	0.324	
Pets	1	166.186	166.186	6.902	9.252	0.716	0.408	
Months on the job	1	186.059	186.059	0.071	0.079	0.802	0.382	
Residuals	18	4175.083	231.949	NA	NA	NA	NA	
Reduced Model								
None								
**Compassion satisfaction**								
Original Model								
Job	2	36.890	18.445	−0.442	4.174	0.803	0.464	
Gender	1	272.382	272.382	6.358	2.164	11.852	0.003	**
Age	1	5.327	5.327	−0.095	1.605	0.232	0.636	
Smoke	1	65.798	65.798	2.521	2.414	2.863	0.108	
Married	1	11.200	11.200	3.757	2.962	0.487	0.494	
Spiritual	1	58.737	58.737	−3.524	2.651	2.556	0.127	
Contact with animals	1	55.696	55.696	−1.518	1.193	2.423	0.137	
Pets	1	123.048	123.048	−6.482	2.912	5.354	0.033	*
Months on the job	1	11.381	11.381	−0.017	0.025	0.495	0.491	
Residuals	18	413.679	22.982	NA	NA	NA	NA	
Reduced Model								
Gender	1	214.520	214.524	5.284	1.75	9.701	0.005	**
Married	1	122.800	122.799	4.719	1.893	5.553	0.027	*
Pets	1	163.990	163.992	−6.906	2.536	7.416	0.012	*
Residuals	25	552.820	22.113	NA	NA	NA	NA	
Secondary traumatic stress								
Original Model								
Job	2	48.772	24.386	−0.925	5.703	0.569	0.576	
Gender	1	0.508	0.508	1.306	2.956	0.012	0.915	
Age	1	5.868	5.868	0.598	2.193	0.137	0.716	
Smoke	1	0.116	0.116	0.504	3.297	0.003	0.959	
Married	1	43.984	43.984	−5.019	4.047	1.025	0.325	
Spiritual	1	5.616	5.616	0.336	3.621	0.131	0.722	
Contact with animals	1	127.098	127.098	−2.081	1.630	2.963	0.102	
Pets	1	14.727	14.727	1.796	3.979	0.343	0.565	
Months on the job	1	64.720	64.720	0.042	0.034	1.509	0.235	
Residuals	18	772.041	42.891	NA	NA	NA	NA	
Reduced Model								
None								
PC1: rMSSD, pNN50, heart rate, and waking saliva cortisol								
Original Model								
Job	2	4.484	2.242	−2.477	1.674	0.607	0.556	
Gender	1	3.125	3.125	−1.456	0.868	0.846	0.370	

Age	1	1.710	1.710	0.783	0.644	0.463	0.505
Smoke	1	14.594	14.594	−1.730	0.968	3.950	0.062
Married	1	0.483	0.483	0.627	1.188	0.131	0.722
Spiritual	1	0.000	0.000	−0.010	1.063	0.000	0.995
Contact with animals	1	0.055	0.055	−0.225	0.478	0.015	0.905
Pets	1	2.113	2.113	0.961	1.168	0.572	0.459
Months on the job	1	1.639	1.639	−0.007	0.010	0.444	0.514
Residuals	18	66.510	3.695	NA	NA	NA	NA
Reduced Model							
None							
PC2: systolic and diastolic blood pressure							
Original Model							
Job	2	0.721	0.361	−1.555	1.134	0.213	0.811
Gender	1	0.041	0.041	−0.200	0.588	0.024	0.879
Age	1	0.058	0.058	−0.250	0.436	0.034	0.855
Smoke	1	1.574	1.574	−0.109	0.656	0.928	0.348
Married	1	3.903	3.903	1.249	0.805	2.301	0.147
Spiritual	1	2.068	2.068	0.772	0.720	1.219	0.284
Contact with animals	1	4.567	4.567	−0.451	0.324	2.692	0.118
Pets	1	0.545	0.545	−0.453	0.791	0.321	0.578
Months on the job	1	0.010	0.010	0.001	0.007	0.006	0.939
Residuals	18	30.534	1.696	NA	NA	NA	NA
Reduced Model							
None							
